# The roles of lncRNAs and miRNAs in pancreatic cancer: a focus on cancer development and progression and their roles as potential biomarkers

**DOI:** 10.3389/fonc.2024.1355064

**Published:** 2024-03-15

**Authors:** Somayeh Jafari, Hossein Motedayyen, Parisa Javadi, Kazem Jamali, Amin Moradi Hasan-Abad, Amir Atapour, Gholamreza Anani Sarab

**Affiliations:** ^1^ Department of Molecular Medicine, School of Medicine, Birjand University of Medical Sciences, Birjand, Iran; ^2^ Autoimmune Diseases Research Center, Kashan University of Medical Sciences, Kashan, Iran; ^3^ Department of Medical Nanotechnology, School of Advanced Medical Sciences and Technologies, Shiraz University of Medical Sciences, Shiraz, Iran; ^4^ Emergency Medicine Research Center, Shiraz University of Medical Sciences, Shiraz, Iran; ^5^ Trauma Research Center, Shahid Rajaee (Emtiaz) Trauma Hospital, Shiraz University of Medical Sciences, Shiraz, Iran; ^6^ Department of Medical Biotechnology, School of Advanced Medical Sciences and Technologies, Shiraz University of Medical Sciences, Shiraz, Iran; ^7^ Cellular and Molecular Research Center, Birjand University of Medical Sciences, Birjand, Iran

**Keywords:** pancreatic ductal adenocarcinoma, cancer progression, RNAs, potential biomarkers, lncRNAs, miRNAs

## Abstract

Pancreatic ductal adenocarcinoma (PDAC) is among the most penetrative malignancies affecting humans, with mounting incidence prevalence worldwide. This cancer is usually not diagnosed in the early stages. There is also no effective therapy against PDAC, and most patients have chemo-resistance. The combination of these factors causes PDAC to have a poor prognosis, and often patients do not live longer than six months. Because of the failure of conventional therapies, the identification of key biomarkers is crucial in the early diagnosis, treatment, and prognosis of pancreatic cancer. 65% of the human genome encodes ncRNAs. There are different types of ncRNAs that are classified based on their sequence lengths and functions. They play a vital role in replication, transcription, translation, and epigenetic regulation. They also participate in some cellular processes, such as proliferation, differentiation, metabolism, and apoptosis. The roles of ncRNAs as tumor suppressors or oncogenes in the growth of tumors in a variety of tissues, including the pancreas, have been demonstrated in several studies. This study discusses the key roles of some lncRNAs and miRNAs in the growth and advancement of pancreatic carcinoma. Because they are involved not only in the premature identification, chemo-resistance and prognostication, also their roles as potential biomarkers for better management of PDAC patients.

## 1 Introduction

**Figure 1:**
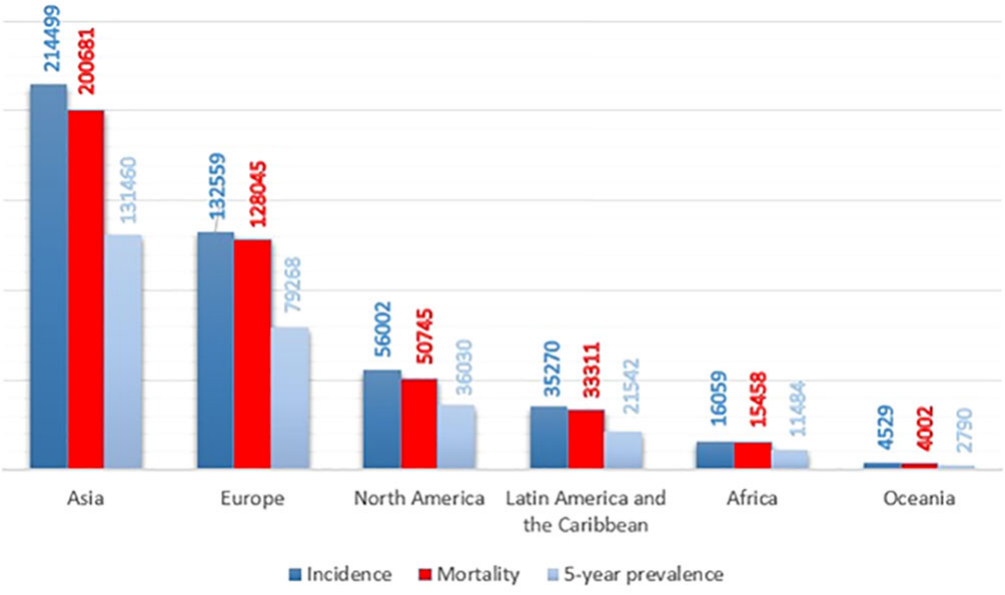
Pancreatic cancer incidence, mortality, and prevalence worldwide by continent in 2020, based on the number of people (1).

Pancreatic ductal adenocarcinoma (PDAC), the vast majority of pancreatic cancer, is among the most infiltrative malignancies, exhibiting a rising prevalence on a global scale. In a high percentage of patients, PDAC is detected in the late and advanced stages, due to the high rate of proliferation and metastasis. Also, this cancer has a poor prognosis, due to the lack of early diagnosis, lack of effective treatment, and chemo-resistance. Hence, PDAC has the shortest overall survival among human cancers. On average, patients with PDAC live six months. Only 7% of patients have an overall survival of 5 years. As a result, the incidence and mortality rates in patients with this cancer are very close (**Figure 1**) (2–4). The pancreas has two exocrine and endocrine parts with different functions. About 90% of pancreatic cancers occur in the exocrine part (5). Various methods, such as surgery, immunotherapy, chemotherapy, and radiotherapy are used to treat pancreatic cancer. Unfortunately, even the use of a combination of these therapies is not effective in PDAC management and is not able to save most patients’ lives. Gemcitabine is a first-line treatment for PDAC with moderate efficacy. Fluorouracil, oxaliplatin, and irinotecan are other common drugs used to treat pancreatic cancer. The combination of these drugs with gemcitabine has yielded promising results. But it has not had much effect on the overall survival and prognosis of the patient (6–10). As a result, the detection of potential biomarkers for early diagnosis, treatment, and prognosis, and then effective strategies are crucial in PDAC therapy (11–13).

**Figure 2:**
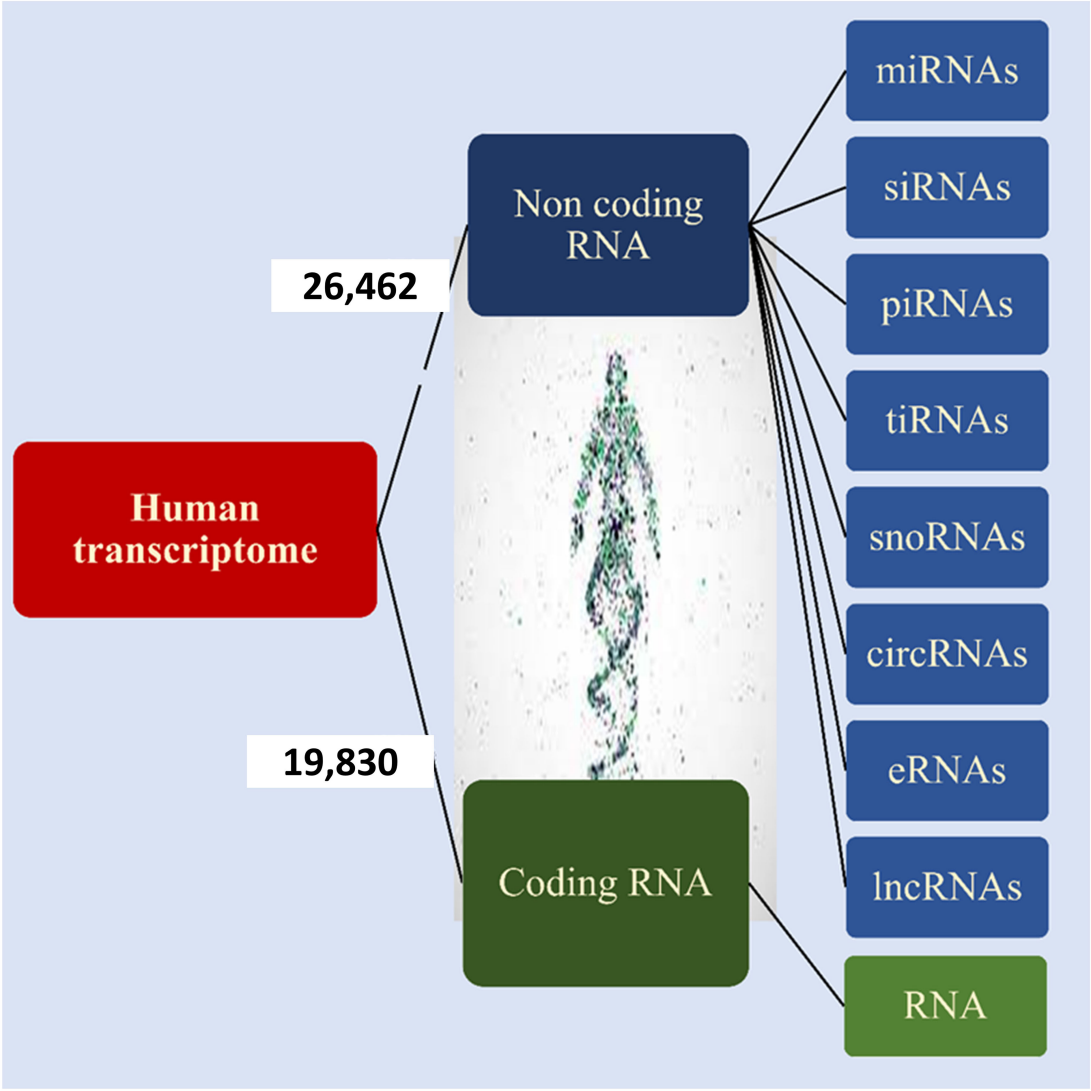
Human transcriptome. Based on the ENCODE project approximately 35% of human genes encode proteins and the remaining 65% of them encode noncoding RNA that is shown in the figure.

According to Ensembl databases, it has been found that approximately 19,830 human genes encode proteins, while 26,462 are non-coding genes (**Figure 2**) (14, 15). These RNAs are called noncoding RNAs (ncRNAs). NcRNAs are grouped into two categories, small noncoding RNAs (<200 bp) and long noncoding RNAs (lncRNAs) (> 200 bp), based on their length. NcRNAs are also categorized based on their function into different groups, including microRNAs (miRNAs), small interfering RNAs (siRNAs), PIWI-interacting RNAs (piRNAs), tRNA-derived stress-induced RNAs (tiRNAs), small nucleolar RNAs (snoRNAs), circular RNAs (circRNAs), enhancer noncoding RNAs (eRNAs) and long noncoding RNAs (lncRNAs) (13, 16, 17). NcRNAs play a vital role in DNA replication, RNA splicing, translation, and epigenetic regulation (18, 19). They are also involved in some cellular processes, such as proliferation, differentiation, metabolism, and apoptosis (16, 17, 20). Various studies have shown that ncRNAs as oncogenes or tumor suppressors play an important role in tumorigenesis of various tissues (16, 17, 21, 22).

In this review among noncoding RNAs, we describe the role of lncRNAs and miRNAs in the development and progression of pancreatic cancer. Various studies have suggested the role of these noncoding RNAs in the initiation, proliferation, progression, and chemo-resistance of pancreatic cancer. Therefore, these noncoding RNAs can be used not only in early diagnosis and prognosis but also for better management of PDAC patients (12, 13, 16, 17, 23).

## 2 lncRNA in PDAC

**Figure 3:**
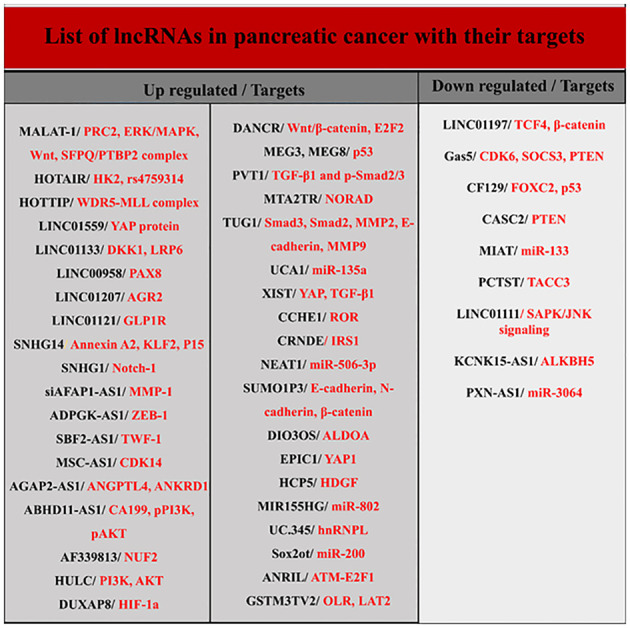
List of lncRNAs and their targets in pancreatic cancer.

LncRNAs are longer than 200 nucleotides and are transcribed by RNA polymerase II. Usually, lncRNAs, like mRNAs, have a 5’ cap and a 3’ polyadenylated tail and are spliced ​​to produce the final version. They are present in both the nucleus and the cytoplasm, but the stimuli that induce their organization in these regions remain unknown (12, 14, 24). Although lncRNAs constitute the majority of transcriptomes, their biological functions are less well known. LncRNAs genes are usually dispersed throughout the genome and their target genes are located very close to them. They are involved in several activities, such as cis- and trans-acting effect and genomic imprinting through various mechanisms, including transcriptional interference, antisense inhibition, and chromatin remodeling complexes. Numerous investigations have proposed that lncRNAs have a significant impact on the development of tumors as either oncogenes or tumor suppressors (**Figure 3**) (12, 23, 25). In pancreatic cancer, different lncRNAs are involved in determining the type of biological behavior and cell signal transduction during the cell cycle, autophagy, apoptosis, EMT, and cancer stem cells (CSCs). They also serve as plausible biomarkers in the diagnosis and therapeutic management of pancreatic carcinoma (12, 21, 23–26). Below, the role of some key lncRNAs in PDAC is discussed.

### 2.1 HOX transcript antisense intergenic RNA

HOTAIR is one of the most important lncRNAs in pancreatic cancer, which is involved in regulating neighboring homeobox (HOX) genes. In a normal cell, HOTAIR through its 5’ and 3’ domains selectively bind to polycomb repressive complex 2 (PRC2) protein complexes and lysine specific histone demethylase 1 (LSD1)/REST corepressor 1 (coREST) ​​/RE1 silencing transcription factor (REST), respectively (25, 27, 28). HOTAIR then cleaves these complexes at the HOXD locus on chromosome 2. This process eventually leads to histone H3K27 trimethylation, histone H3K4 demethylation, and alterations in the transcriptional patterns of certain genes implicated in cell proliferation and metastasis (27, 28).

HOTAIR is expressed in high levels in pancreatic cancer tissues compared to adjacent normal cells and significantly increases the cell’s ability for proliferation, invasion, and metastasis (25, 28). It has been demonstrated that HOTAIR expression in pancreatic cancer cell lines via the Wingless (Wnt)/β-catenin signaling pathway downregulates N-cadherin, β-catenin, vimentin, cyclinD1, c-Jun, Lymphoid enhancer binding factor 1 (LEF1) and cellular myelocytomatosis oncogene (c-myc), upregulate E-cadherin and inhibit epithelial–mesenchymal transition (EMT) (29, 30). Also, after radiotherapy, HOTAIR down-expression by overexpression of Wnt inhibitory factor 1 (WIF-1) and autophagy related 7 (ATG7) reduces radio-resistance and cell proliferation and induces apoptosis in pancreatic cancer patients. HOTAIR activates the hexokinase-2 enzyme in pancreatic cancer cells. Activation of this enzyme promotes cell glucose uptake and enhances the value of energy carriers such as ATP. As a result, HOTAIR is involved in increasing cellular energy levels and growth induction in pancreatic cancer cells. In addition, HOTAIR is caused resistance to TRAIL-induced apoptosis by modulating EZH2-mediated histone H3 lysine 27 trimethylation (H3K27me3) in pancreatic cancer cells (30, 31).

HOTAIR also regulates the expression of miRNAs and modulates key signaling pathways involved in pancreatic cancer progression. For example, the activity of MiR-663b is suppressed by HOTAIR. MiR-663b binds to the 3’ untranslated region (3’UTR) region of insulin Like growth factor 2 (IGF2) mRNA and inhibits its expression and cell proliferation in the following. HOTAIR increases proliferation in pancreatic cancer cells via miR-663b inhibition and overexpression of IGF2 (30, 32). The function of miR-613 in pancreatic cancer is also tumor suppression by targeting the neurogenic locus notch homolog protein 3 (notch3). HOTAIR is also able to modulate the activity of this microRNA. One study reported that HOTAIR silencing with RNAi downregulates neurogenic locus notch homolog protein 3 (Notch3) by down-expression of miR-613 and suppresses tumor growth (30, 32). MiR-34a is another direct target of HOTAIR that is downregulated through enhancer of zeste 2 polycomb repressive complex 2 subunit (EKH2) mechanisms during pancreatic cancer. HOTAIR activates the Janus kinase 2 (JAK2)/signal transducer and activator of transcription 3 (STAT3) signaling pathway by suppressing the miR-34a expression, increasing proliferation, invasion, and migration in CSCs (30, 33).

The rs200349340 and rs4759314 polymorphisms of the HOTAIR gene are associated with pancreatic cancer (34, 35). As a prognostic biomarker, HOTAIR expression in the late stages of pancreatic cancer indicates poor patient survival and cancer progression. HOTAIR is also important as a diagnostic biomarker in pancreatic cancer patients. Detection of this noncoding RNA in salvia is a biomarker with a sensitivity of 78.20% and specificity of 85.60% in diagnosing pancreatic cancer. Also, an increased level of HOTAIR in serum is associated with the upper stage of the pancreatic tumor (21, 33).

### 2.2 HOXA transcript at the distal tip

HOTTIP is another HOX-dependent lncRNA that is transcribed from the 5’ end of the HOXA locus. HOTTIP controls the expression of several genes by interaction with polycomb repressive complex 2 (PRC2) and mixed lineage leukemia protein-1 (MLL1)/WD repeat domain 5 (WDR5) in this locus (36, 37). This lncRNA is significantly expressed in pancreatic cancer tissues and cell lines compared to normal (37). HOTTIP has been shown to control the proliferation, apoptosis, and migration of pancreatic cancer cells by regulating several HOX genes, including HOXA13, HOXA10, HOXB2, HOXA11, HOXA9, and HOXA1. Inhibition of HOTTIP decreases cell proliferation, reduces cell invasion through suppression of EMT and enhances the antitumor effects of gemcitabine in pancreatic cancer *in vitro* and *in vivo* (36, 37).HOTTIP has been reported to increase proliferation in pancreatic cancer cells by activating the WNT/beta-catenin signaling pathway. HOTTIP downregulation enhances apoptosis in cancer cells by pro-apoptotic proteins overexpression, caspases activation, and inhibition of the phosphatidylinositol 3-kinase (PI3K)/Ak strain transforming (AKT)/mechanistic target of rapamycin Kinase (mTOR) signaling pathway (38).

Like other lncRNAs, the HOTTIP function is associated with some ncRNAs. MiR-497-5p has suppressive tumor properties and can repress HOTTIP expression. Augmented CircRTN4 levels are commonly seen in primary pancreatic tumors. CircRTN4 enhances the growth and metastasis of cancer cells by inhibiting miR-497-5p and increasing HOTTIP expression (39). The miR-137 is also a downstream target for HOTTIP. MiR-137 down-expression by HOTTIP is involved in chemo-resistance. HOTTIP silencing or miR-137 upregulation promotes the susceptibility of pancreatic cancer cells to cisplatin, reducing proliferation and increasing apoptosis in these cells (36). However, HOTTIP is supposed to be one of the predictors of response to chemotherapy in pancreatic cancer. HOTTIP also determines the pancreatic cancer cells’ resistance to gemcitabine, by overexpression of HOXA13. HOXA13 upregulation triggers the growth and metastasis of cancer cells and determines the poor survival of patients (30).

One study found that populations with rs1859168 A> C polymorphism in the HOTTIP encoding gene were at a lower risk for pancreatic cancer (40).

### 2.3 H19

H19 is the first lncRNA to be distinguished in human pathology. Commonly, H19 is highly expressed during embryonic development. But, after birth, its expression is suppressed (41, 42). Some studies have reported H19 re-expression in some types of cancer, such as the esophagus, colon, liver, and bladder (41–43). In pancreatic cancer, not only H19 is overexpressed in tumor tissue and cell lines, but it is also positively associated with the ability of tumor invasion and migration (43, 44). This lncRNA is the antagonist of a let-7 miRNA and promotes the development of elevated mobility group AT-Hook 2 (HMGA2)-dependent EMT (44). The research studies have demonstrated that DNA-based therapies against the H19 gene sequence alone or combined with gemcitabine can enhance the therapy of pancreatic carcinoma. H19 also determines the prognosis of pancreatic cancer (42, 44).

H19 function and expression are inversely related to miR-675. Upregulation of miR-675 arrests the cell cycle in the S phase and prevents cancer cell proliferation. H19 promotes cancer progression through the H19/miR-675/E2F transcription factor 1 (E2F-1) signaling pathway. The H19/miR-675 axis is also involved in the invasion and metastasis of cancer cells by targeting some mediators such as suppressors of cytokine signaling 5 (SOCS5) and regulating STAT3 function. MiR-675 is a diagnostic biomarker in pancreatic cancer (45–47).

Usually, H19 co-regulates with PFTAIRE protein kinase 1 (PFTK1) in pancreatic cancer. If H19 is inhibited, PFTK1 overexpression via Wnt/β-catenin signaling reverses the effects of H19 on cell proliferation and invasiveness. MiR-194 is a negative regulator of PFTK1 expression. Upregulation of miR-194 reduces the PFTK1 expression and attenuates cell proliferation (45, 46).

### 2.4 Plasmacytoma variant translocation 1

PVT1 is also an lncRNA with an oncogenic role in many malignant tumors (48, 49). In addition to H19, PVT1 has been shown to increase EMT in pancreatic cancer tissue by activating the TGF-β/SMAD signaling pathway and inducing proliferation and metastasis by inhibiting SERPINE1 mRNA binding protein 1 (SERBP1). PVT1 also regulates the development and progression of pancreatic cancer (48, 50–52).

During pancreatic cancer, severe hypoxia occurs in the tumor microenvironment. It has been shown that PVT1 upregulates simultaneously with hypoxia inducible factor 1 subunit alpha (HIF-1α) in hypoxic-cancer cells. PVT1 can overexpress and activate HIF-1α by binding to its promoter region at two levels of transcription and post-translation, respectively. PVT1 has also been revealed to bind directly to a tumor suppressor microRNA called miR-519d-3p, and inhibit its expression. HIF-1α is one of the downstream targets of miR-519d-3p. Suppression of miR-519d-3p by PVT1 increases the HIF-1α expression, triggers the Wnt/β-catenin signaling pathway, and promotes the growth and proliferation of pancreatic cancer cells. Given the role of the PVT1/HIF-1α regulatory loop in the progression of pancreatic cancer, this path may be one of the promising therapeutic goals in managing pancreatic cancer (53, 54).

One of the reasons for poor prognosis in patients with pancreatic cancer is chemo-resistance. PVT1 can stimulate the Wnt/β-catenin signaling pathways and autophagy through the function of miR-619-5p in the miR-619-5p/autophagy-related 14 (ATG14) and miR-619-5p/pygopus homolog 2 (Pygo2) axes, causing chemo-resistance promotion in pancreatic cancer (50, 55). On the other hand, studies have shown that PVT1 is involved in the induction of gemcitabine resistance by binding to the EZH2 and forming the PVT1/EZH2 complex in pancreatic cancer. Histone acetyltransferase 1 (HAT1) is one of the upstream mediators of PVT1, which determines the cancer cells’ resistance to gemcitabine. HAT1 triggers more binding of bromodomain-containing 4 (BRD4) to the PVT1 gene promoter and causes further PVT1 expression. HAT1, on the other hand, prevents the ubiquitination of EZH2 and increases its Lifetime in the cell. Due to the regulatory role of HAT1 in the PVT1 expression, HAT1 can be considered one of the therapeutic targets for reducing the oncogenic effects of PVT1 on pancreatic cancer cells. Also, targeting each of HAT1, PVT1, miR-619-5p, and EZH2 individually or in combination may enhance the cancer cells’ resistance to gemcitabine and increase the effects of chemotherapy and prognosis in patients (31, 56, 57). Besides, studies have shown that PVT1 regulates gemcitabine sensitivity in pancreatic cancer via miR1207 and thus plays a role in drug resistance.

Detection of PVT1 in saliva is a diagnostic biomarker with 96.4% sensitivity and 63.6% specificity in pancreatic cancer (21). Also, increasing the amount of PVT1 with MALAT1 and HOTTIP in serum has been suggested to predict the effect of gemcitabine in pancreatic cancer (21).

### 2.5 Metastasis-associated lung adenocarcinoma transcript 1

MALAT-1 has increased in many types of cancers, including pancreatic cancer and pancreatic cell lines (58–60). This lncRNA is associated with characteristics such as tumor size, clinical phase, invasion ability, metastasis to lymphatic nodes, low overall survival, and poor prognosis in patients with cancer (21, 59, 60). MALAT-1 can regulate Kirsten rat sarcoma virus (KRAS) expression by means of competitive inhibition, thereby increasing cell proliferation in pancreatic cancer (61, 62). Knockout of MALAT-1 arrests the cell cycle in the G2/M phase, inhibits EMT, suppresses N-myc downregulated gene-1 (NORG-1), and suppresses the growth and invasiveness of neoplasm cells (62). Detection of MALAT1 in pancreatic tissue is a diagnostic biomarker with 66% sensitivity and 72% specificity for pancreatic cancer (21). Overall, this lncRNA acts as a progressive factor for pancreatic cancer and is recommended to be contemplated as a target in the therapy of pancreatic neoplasia (62).

### 2.6 Highly upregulated in liver cancer

HULC is also a cancer-associated lncRNA that regulates proliferation, migration, viability, and invasion in pancreatic cancer (21, 63, 64). Also, HULC is highly expressed in advanced pancreatic tumors. High expression of HULC activates the PI3K/AKT signaling pathway by reducing the expression of miR-15a and causing tumorigenesis (64, 65). Higher expression of HULC is associated with poor prognosis in patients with pancreatic cancer (63, 64). HULC has been identified as a perfect diagnostic biomarker in pancreatic cancer, so its detection in serum with 93.33% sensitivity and 96.67% specificity can detect the size and stage of the tumor as well as vascular invasion (21).

### 2.7 Other lncRNAs in PDAC

Other lncRNAs have also been detected in pancreatic cancer (**Figure 3**). For example, growth arrest-specific 5 is one of these lncRNAs significantly down-expressed in pancreatic cancer tissue and cell lines and is involved in cell proliferation and cell cycle regulation (21, 66, 67). Expression of another lncRNA called ENST00000480739 also manifests a noteworthy decrease in pancreatic neoplastic tissue in contrast to surrounding non-malignant tissue, and its expression levels are negatively associated with tumor advancement and inferior survival outcomes in patients afflicted with pancreatic cancer (21).

## 3 miRNAs in PDAC

miRNAs are the most widely known family of ncRNAs. Usually, miRNAs are transcribed by the enzyme RNA polymerase II. The primary transcripts are called pri-miRNA that mature through Drosha and Dicer-dependent or non-Drosha-Dicer-dependent pathways and produce functional versions of miRNA. Of course, miRNAs can also be produced as intermediate products during snoRNA, tRNA, or Y-RNA synthesis.

The human genome contains myriad miRNAs that govern a substantial portion of the human transcriptome. They have complex functions and affect a wide range of biological pathways (68–70). Multiple miRNAs can affect a specific mRNA transcript expression. Besides, a particular miRNA may also regulate the expression of different mRNAs in different signaling pathways. Dysregulation of miRNAs stimulates tumorigenesis in the pancreatic tissue. Dysregulated miRNAs can be classified into two categories, oncogene (oncomiR) or tumor suppressor (tsmiR), based on their function in pancreatic carcinogenesis. Many studies have suggested the role of miRNAs in cell proliferation, apoptosis, invasion, metastasis, and chemo-resistance in pancreatic cancer (71–73).

**Figure 4:**
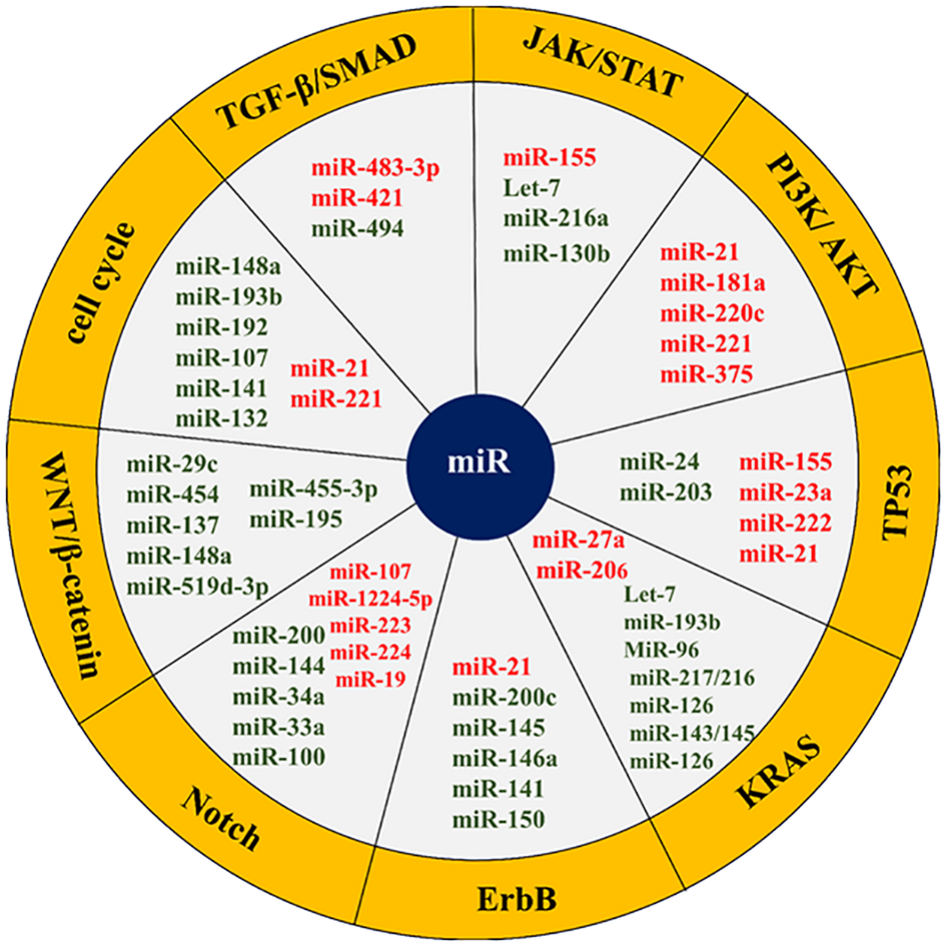
List of some microRNAs related to key cell signaling pathways in pancreatic cancer. The red colors represent an oncogene and the green color shows tumor suppressors. The microRNAs related cell signaling pathways are shown in the yellow margin.

MicroRNAs play a hopeful role as potential biomarkers not only in early diagnosis and prognosis, but also in the treatment of pancreatic cancer. Identifying microRNAs that are most commonly dysregulated during pancreatic cancer and their associated signaling will be very promising in better understanding and clinical management of pancreatic cancer (71–73). Accordingly, the miRNAs frequently misregulated in pancreatic cancer and have a role in the key mediator regulation of main signaling pathways are described below (**Table 1**, **Figure 4**).

**Table 1:** Types of miRNA in pancreatic cancer.

RNAs	Gene sizes (base on GRCh38/hg38)	Genomic locations	Expressions	Roles	Targets	Roles in pancreatic cancer	Ref.
miR-124	85 -109 bases	8p23.1, 8q12.3, 20q13.33	Down regulate	Tumor suppressor	RAC1	Proliferation, invasion, metastasis	(74–77)
miR-203	86-110 bases	14q32.33	Down regulate	Tumor suppressor	BIRC5, CAV1 Bmi-1, CKAP2, LASP1, WASF1, ASAP1, SNAI1/2, RUNX2, ZEB1/2, AKT2	Cell cycle progression, apoptosis, EMT	(78–80)
miR-143	25,990 bases	5q32	Down regulate	Tumor suppressor	GET1, GET2, KRAS RREB1, COX2, TAK1	Proliferation, invasion, metastasis	(21, 81–83)
miR-126	85 bases	9q34.3	Down regulate	Tumor suppressor	E2F2, c-Myc, KRAS, MAPK, STAT3, ADAM9	Proliferation	(84)
Let-7	–	9q12	Down regulate	Tumor suppressor	IGF family, E2F2, c-Myc, KRAS, MAPK, STAT3, HMGA1, HMGA2, IGF2BP1, IGF2BP3, SOCS3, RRM2, N-adherin/ZEB1, NF2, TRIM71, LIN28	Proliferation, Drug resistance	(21, 69, 85–88)
miR-34a/b	84-33,819 bases	1p36.22	Down regulate	Tumor suppressor	TP53, Bcl-2, Notch1/2/3, Snail1, CCND1, E2F1, E2F3, BCL-2, C-MYC, SNAIL1, CDK6, SIRT1, SMAD3	apoptosis, DNA repair, Cell cycle progression, angiogenesis	(21, 89–92)
miR-200a	78-95 bases	1p36.33, 12p13.31	Down regulate	Tumor suppressor	EP300, ZEB1, SIPI1, PTEN, MT1-MMP, ZEB2, SOX2, E-cadherin, Vimentin	EMT	(21, 58, 69, 88, 93–96)
miR-146a	383 bases	5q33.3	Down regulate	Tumor suppressor	IRAK-1, EGFR	Invasion	(93, 97–99)
miR-96	78 bases	7q32.2	Down regulate	Tumor suppressor	KRAS, AKT	Tumor growth, invasion	(69, 93, 100–102)
miR-21	3,434 bases	17q23.1	Up regulate	Oncogene	PTEN, EGFR, HER2/neu, PDCD4, Bcl2, TIMP2, TIMP3, CDK6, CDKN1A, IL-6R, FAS, TPM1, APAF1, SOCS5	Proliferation, cell division	(21, 69, 75, 93, 103, 104)
miR-221/222	110 bases	Xp11.3	Up regulate	Oncogene	CDKN1B, PUMA, PTEN, Bim, MMP-2, MMP-9, TIMP-2, P27kip1, P57kip2, KIT, CDKN1C	Cell cycle progression	(21, 69, 105–108) (109)
miR-192	110 bases	11q13.1	Up regulate	Oncogene	SIP1, Cell cycle regulatory genes	Proliferation, migration, apoptosis, Cell cycle progression	(110, 111)
miR-424	98 bases	Xq26.3	Up regulate	Oncogene	SOCS6	Cell proliferation, migration	(112–114)
miR-208a	80 bases	14q11.2	Up regulate	Oncogene	CDH1	EMT	(115–117)
miR-155	65 bases	21q21.3	Up regulate	Oncogene	TP53INP1, SOCS1, SOCS3	apoptosis	(21, 118)
miR-10	110 bases	17q21.32, 2q31.1	Up regulate	Oncogene	HOXB8, HOXA1, TFAP2C, HOXA3	Invasion, metastasis	(21, 119, 120)
miR-196a-2/196	110 bases	12q13.13	Up regulate	Oncogene	HOXB8, ANXA1, HMGA2	–	(93, 121)
miR-375	68 bases	2q35	Up regulate	Oncogene	PDK1, 14-3-3zeta	Proliferation, apoptosis	(122–124)
miR-210	110 bases	11p15.5	Up regulate	Oncogene	HOXA1, FGFRL1, HOXA9, COX10, E2F3, RAD52, ACVR1B, MNT	Regulation of interaction between cancer cells and stellate cells	(21, 125–127)
miR-301a	86 bases	17q22	Up regulate	Oncogene	Bim, NKRF	Proliferation, metastasis	(128–130)
miR-421	85 bases	Xq13.2	Up regulate	Oncogene	PANCREATIC CANCER4/Smad	Proliferation, colony formation	(131–133)
miR-16-1/2	89 -105 bases	13q14.2, 3q25.33	Up regulate	Oncogene	Bcl2l1, Naip5, fgfr2, mybl2	Apoptosis, angiogenesis	(134, 135)
miR-15-a/b	85-98 bases	13q14.2, 3q25.33	Up regulate	Oncogene	Bcl2l1, Naip5, fgfr2, mybl2, WANT3A, FGF7, BMI-1	Apoptosis, angiogenesis	(69, 135–137)

The transforming growth factor-beta (TGF-β)/mothers against DPP homolog (SMAD) signaling pathway is a key signaling pathway in pancreatic cancer. SMAD4 in the TGF-β/SMAD signaling pathway is a regulator of TGF-β that is involved in cell growth and proliferation. SMAD4 function is eliminated in most pancreatic cancer patients. miR-494 plays a tumor-suppressive role in pancreatic cancer and inhibits EMT invasion and migration of cancerous cells via TGF-β/SMAD and hepatocyte growth factor (HGF)/MET signaling pathways. Increasing the miR-494 expression levels in these cells *in vitro* and *in vivo* inhibits its direct targets (c-Myc and sirtuin 1 (SIRT1)) expression, triggers apoptosis, arrests the cell cycle in the G1 phase, and stops cell proliferation. Pancreatic cancer patients with lower miR-494 expression values have larger tumor sizes, lymphatic invasion, and poor prognosis. Loss of SMAD4, which occurs in most patients’ pancreatic cancer, is supposed to reduce the expression of miR-494 (71, 138, 139). In addition to miR-494, the function of two other miRNAs called miR-421 and miR-483-3p is related to SMAD4. MiR-421 is an oncomiR and a key regulator of SMAD4 expression. This miRNA is strongly overexpressed, while the SMAD4 expression is subsequently downregulated in pancreatic cancer patients. It has even been shown that the addition of ectopic miR-421 to pancreatic cancer cell lines considerably reduces the SMAD4 expression in these cells, resulting in cell proliferation and cell invasion (131, 132). MiR-483-3p is also upregulated in the early stages of pancreatic carcinogenesis. Detecting miR-483-3p in serum is one of the most potent biomarkers in the diagnosis and prognosis of pancreatic cancer. Its plasma levels are also involved in pancreatic cancer differentiation from intraductal papillary mucinous neoplasms (IPMNs) (140, 141).

The JAK/STAT signaling pathway is another key cellular pathway in pancreatic cancer that plays a role in cell growth, cell division, cell death, and inflammation. Let-7 microRNA is a tsmiR that affects JAK/STAT signaling axis and regulates cell cycle, proliferation, metabolism, and apoptosis in the pancreatic cell. Let-7 expression is reduced in pancreatic cancer cells compared to adjacent normal cells. The reinstatement of Let-7 expression levels in pancreatic neoplastic cells by targeting KRAS results in the restraint of cell proliferation but does not prevent tumor cell growth. Let-7 re-expression in pancreatic cancer cells also enhances the cytoplasmic expression of cytokine signaling 3 (SOCS3), inhibits STAT3 activation in the JAK-STAT pathway, stops cell proliferation, and increases apoptosis. In addition, Let-7 is a key regulator of the immune response in many human cancers through its association with toll-like receptors and mediation of cytokine expression. Therefore, this microRNA has been considered as a new agent in immuno-cancer therapy (85, 86, 142). Apart from let7, the function of other microRNAs such as miR-155, miR-216a, and miR-130b is related to the JAK-STAT signaling pathway. MiR-155 also increases proliferation and invasion in pancreatic cancer cells by inhibiting the suppressor of cytokine signaling 1 (SOCS1). MiR-216a is a conserved and tissue-specific microRNA that plays a pivotal role in the regulation of pancreatic function. In pancreatic cancer, this miRNA down-expresses. MiR-216a down-regulation is usually accompanied by the size and mass reduction of pancreatic beta cells, a decrease in insulin secretion, and a diminution in the acinar cells’ function. Inhibition of miR-216a also enhances apoptosis and reduces pancreatic cell proliferation. MiR-216a is involved in the progression of pancreatic cancer through the two-axis, including miR-216a/tetraspanin-1 (TSPAN1)/integrin alpha-2 precursor (ITGA2) and LINC01133/miR-216a-5p/translationally-controlled tumor protein (TPT1). Given that miR-216a circulating levels increase in pancreatic cancer patients, it has been suggested that miR-216a could serve as a promising biomarker for the clinical assessment and management of pancreatic cancer (143–145).

The PI3K/AKT signaling pathway can be targeted by several miRNAs, such as miR-21, miR-181a, miR-220c, miR-221, and miR-375. miR-221 is a proto-oncogene that its overexpression increases proliferation and invasion, inhibits apoptosis, and induces chemo-resistance in pancreatic cancer (64, 65). This miRNA is an important biomarker in the study of invasion and metastasis. MiR-221 targets matrix metalloproteinases 2 (MMP2) and matrix metalloproteinases 9 (MMP9) genes, which are closely related to cell migration and invasions. Besides, miR-221 has other targets such as PTEN, which negatively regulates cell proliferation and survival by counteracting PI3K signaling (105–107). MiR-21 upregulation causes gemcitabine resistance and triggers pancreatic cancer cell malignancy through p85α (the PI3K regulatory subunit). MiR-21 also plays an oncogenic role in other cancers by targeting the forkhead box O1 (FOXO1), protein tyrosine phosphatase non-receptor type 14 (PTPN14), and PTEN (89, 103, 104, 146).

The roles of miR-192-5p and miR-192-3p in pancreatic cancer involve binding to targets within critical cellular signaling pathways such as PI3K/AKT/mTOR and mitogen-activated protein kinase (MAPK). This binding inhibits pathway activity, leading to reduced cancer cell proliferation, migration, and apoptosis. Furthermore, the correlation between the levels of miR-192-5p and miR-192-3p and DNA methylation at proximal CpG sites underscores their epigenetic regulation in pancreatic cancer. Dysregulated DNA methylation patterns can impact miRNA expression and downstream target genes involved in tumorigenesis. Additionally, these microRNAs are associated with specific mRNAs, including PLAU, CAV1, and CDH2, affecting processes such as cell adhesion and metastasis. Moreover, miR-194-3p and miR-192-3p have been identified as the most discriminatory microRNAs for pancreatic cancer among all TCGA cancers, indicating their potential as biomarkers for diagnosis, prognosis, and therapeutic stratification. Leveraging the unique expression profiles of these microRNAs may enable clinicians to enhance early detection and personalize therapy for pancreatic cancer patients, ultimately leading to improved clinical outcomes (147–151).

Some microRNAs, such as miR-155, miR-203, miR-23a, miR-222, miR-21, and miR-24 are engaged in the TP53 signaling pathway and activation of apoptosis. MiR-155 can induce apoptosis and inhibit cell proliferation in some pancreatic cell lines. This miRNA promotes pancreatic cancer progression by reducing the expression of some genes, such as the TP53-induced nuclear protein 1 gene (TP53INP1). TP53INP1 is involved in increasing TP53 activity and suppressing tumorigenesis (93, 152). MiR-203 expression is reduced in pancreatic cancer cells. This miRNA inhibits the expression of Fibroblast Growth Factor 2 (FGF2) and decreases proliferation, apoptosis, invasion, and migration in the disease (78, 153). The regulatory role of miR-21 in the modulation of essential genes implicated in programmed cell death, commonly known as apoptosis, has been demonstrated. The expression of miR-21 in malignant pancreatic cells is induced by epidermal growth factor (EGF), enhancing proliferation, inhibiting apoptosis, and promoting cell cycle progression. The miR-21 is raised as a diagnostic and prognostic biomarker in pancreatic cancer and is associated with poor prognosis (103, 154).

KRAS signaling pathway represents one of the main signaling pathways in the human cells that in more than 90% of cases acquire gain of function mutation in the early stages of pancreatic carcinogenesis. This pathway is responsible for the proliferation, cell cycle progression, adhesion, migration, apoptosis, and cytoskeletal changes in the cell. Many miRNAs are involved in pancreatic cancer KRAS function regulating, including Let-7, miR-193b, miR-206, MiR-96, miR-217/216, miR-126, miR-143/145, miR-3923, miR-217, miR-126, and miR-27a. Let-7 binds to the 3-UTR region in KRAS mRNA and suppresses its expression. Ectopic expression of Let-7 in pancreatic cancer cells suppresses KRAS expression (71, 155). MiR-193 also has tumor suppressor function and binds to the 3′-UTR ends of KRAS, suppressing the expression of KRAS and inhibiting pancreatic cancer cells proliferation and invasion. MiR-206 can inhibit KRAS and KRAS-induced NF-κB transcriptional activity, thereby reducing angiogenesis and inflammation of pancreatic cancer. MiR-96, unlike bladder, lung, prostate, hepatocellular, and colorectal cancers, is tsMIR in pancreatic cancer and prevents its progression by suppressing KRAS expression. MiR-217/216 also directly targets KRAS and thereby counteracts pancreatic tumor growth. Usually, the expression of miR-217/216 in normal pancreatic cells is higher than in cancerous cells (72, 155).

The erythroblastic oncogene B (ErbB) signaling pathway is a key cellular cascade responsible for regulating cell physiological events. In this path, ErbB1 (epidermal growth factor receptor/EGFR) and especially ErbB2 (HER2/neu) are more important in pancreatic cancer. ErbB1 is mutated in more than 90% of patients with pancreatic cancer and enhances proliferation, angiogenesis, and metastasis in this cancer. Some studies point to the role of ErbB1 in the induction of miR-21 expression. It has also been suggested that miR-200c indirectly affects EGFR expression by regulating mitogen-inducible gene 6 (MIG6) expression. MiR-145 overexpression is followed by inhibition of the exportin 1 (XPO1) gene and downregulates target genes such as EGFR, C-MYC, MMP1, and others. On the other hand, miR-146a upregulates the ErbB1. Neuropilin-1 (NRP-1) is a non-tyrosine kinase receptor that interacts with many cellular pathways, including the ErbB signaling pathway (18, 156). Neuropilin-1 (NRP-1) is negatively regulated by miR-141, a potential biomarker in pancreatic cancer management. ErbB2 overexpression has been observed in 0-82% of pancreatic cancer cases. MiR-150 is also elevated during pancreatic cancer. Various studies have shown a correlation between miR-150 and ErbB2 signaling (72, 157, 158).

The Notch signaling pathway is among the most exceedingly preserved pathways in pancreatic cells that over-activates during pancreatic cancer. This path plays a control role in most physiological processes in the cell and is regulated by numerous miRNAs. MiR-34a is a tumor suppressor microRNA that is down-expressed during pancreatic cancer. Restoring the expression of miR-34a in the cell declines Notch-1/2 expression and impedes the progression of pancreatic cancer. MiR-34a has also been shown to play a role in pancreatic cancer stem cell self-renewal through direct regulation of the Notch pathway (159–163). MiR-1224-5p via miR-1224-5p/ELF3 axis affects PI3K/AKT/Notch signaling pathways in pancreatic cancer cells and causes malignant and aggressive behaviors. Some studies indicate the role of miR-200 in inhibiting Notch signaling pathway components such as mastermind-like coactivators Maml2, Maml3, and jagged canonical Notch ligand 1 (JAG1) and reducing cell proliferation and EMT. MiR-144 has been shown to suppress Notch1 expression in pancreatic cells by binding to the 3′UTR region of Notch1 mRNA (159–163). MiR-223 also downregulates Notch1 in pancreatic tissue. This microRNA plays an important role in regulating motility, migration, invasion, proliferation, and apoptosis in pancreatic cancer cells. In this cancer, inhibition of miR-223 decreases metastasis and suppresses tumor progression. In mucinous cystic neoplasms (MCNs) of the pancreas, miR-224 binds to the 3′UTR region of JAG1 mRNA and regulates its expression. Therefore, miR-224 suppresses Notch signaling by repressing JAG1 expression. In addition to the above, miR-33a, miR-19, miR-8, miR-100, miR-37, and miR-107 are other microRNAs that interact with the Notch signaling pathway (159–163).

The WNT/β-catenin signaling pathway is also one of the highly conserved cellular pathways involved in a wide range of cellular functions. Dysregulation of this signaling pathway induces proliferation, reduces apoptosis, and pancreatic tumorigenesis. Many miRNAs are involved in the regulation of the signaling cascade in normal or cancerous cells. MiR-455-3p inhibits the Wnt/β-catenin signaling pathway via phospholipid-lysophospholipid transacylase (TAZ) and suppresses pancreatic cancer progression. According to the miR-455-3p tumor suppressor role in pancreatic cancer, the miR-455-3p/TAZ/Wnt axis can be a potential therapeutic target in the management of this disease (164). MiR-195 is a tumor suppressor that inhibits the development and progression of pancreatic cancer. Upregulation of miR-195 prevents the proliferation and invasiveness through the fatty acid synthase/Wnt signaling pathway in pancreatic cancer (165). MiR-148a also suppresses the invasion and metastasis of pancreatic cancer by targeting Wnt10b and WNT signaling paths. MiR-148a is usually decreased in individuals affected by this malignancy and is correlated with an unfavorable prognosis (166). MiR-29c down-expresses during pancreatic cancer and directly modulates WNT upstream regulators, including FRAT regulator of WNT signaling pathway 2 (FRAT2), frizzled cass receptor 5 (FZD5), lipoprotein receptor-related protein 6 (LRP-6) and frizzled class receptor 4 (FZD4). Suppression of miR-29c expression by TGF-β activates WNT signaling (167). Some studies have shown that long non-coding RNA00261 (Linc00261) inhibits pancreatic cancer progression by regulating the miR-552 5p/Forkhead Box O3 (FOXO3) axis (168). TSPAN1 is also overexpressed in pancreatic cancer via family members with sequence similarity 83 (FAM83A) in the canonical WNT-Catenin Beta 1 (CTNNB1) signaling pathway. Patients with elevated TSPAN1 have poor overall survival. Both TSPAN1 and FAM83A are direct targets of miR-454 (169). Each of the fatty acid synthase/Wnt, miR-148a/Wnt10b, Linc00261/miR-552-5p/FOXO3, and miR-454/FAM83A/TSPAN1 axes can be valuable biomarkers and potential therapeutic targets in pancreatic cancer. The function of other microRNAs, such as miR-137 and miR-519d-3p, is also related to the WNT/β-catenin signaling pathway (54, 170).

The cell cycle with the cdk/cyclins complex regulates many cellular processes. This signaling pathway is also up/downregulated by many microRNAs. MiR-148a is one of the miRNAs with antitumor properties and is down-expressed during pancreatic cancer. This microRNA prevents invasion and metastasis by reducing the cyclin D1, C-myc, and β catenin expression in pancreatic cancer cells. MiR-148a also dysregulates the expression of various CDK/cyclin complexes and alters cancer cell phenotype by targeting cell division cycle 25B (CDC25B) (71, 171). MiR-221 upregulation can affect the CDKN1B gene expression and promote pancreatic cancer. MiR-222 affects many key cell cycle inhibitors, such as p57 and p27, and promotes cell cycle progression. Contrariwise, miR-107 targets cyclin D1-dependent kinase and inhibits cell cycle progression. MiR-107 appears to be silenced during pancreatic cancer (68, 71, 73, 172). MiR-21 enhances radio-resistance by affecting cell cycle checkpoints (173). MiR-132 increases proliferation and decreases apoptosis in pancreatic cancer cells by cyclin-D1 downregulation and acting on other important cellular mediators such as caspase-3 or -9 (174). MiR-193b interferes with the KRAS signaling pathway, causing cell cycle break and G1 phase arrest. MiR-192 also overexpressed cyclin D1, CDC2, cyclin D2, and cyclin-dependent kinase 4 (CDK4) and promotes G1 to S-phase cell cycle transition (71, 73, 172). MiR-141 is a negative regulator of NRP-1. The miR-141 inhibitory effect on NRP-1 causes cell cycle arrest in G0/G1 phase, upregulates p27 and downregulates cyclin E/CDK2, suppresses cell proliferation and tumor growth, reduces cell migration by inhibiting EMT, and prevents liver metastasis. MiR-141/NRP-1 axis is one of the potentially valuable targets in pancreatic cancer treatment (158).

## 4 Aggressive nature and metastatic spread of PDAC

Pancreatic ductal adenocarcinoma (PDAC) is notorious for its aggressive behavior and propensity for metastasis, significantly impacting patient prognosis and treatment outcomes. As one of the deadliest malignancies, PDAC often manifests insidiously, with nonspecific symptoms and late-stage diagnosis exacerbating its aggressive nature. By the time of diagnosis, many patients already harbor locally advanced or metastatic disease, posing challenges for effective management. The aggressive biology of PDAC is characterized by rapid tumor growth, with tumors demonstrating rapid proliferation and invasive growth patterns, infiltrating surrounding pancreatic tissues and adjacent organs, contributing to early local invasion and metastatic dissemination. Additionally, PDAC tends to metastasize early, with cancer cells disseminating to regional lymph nodes, liver, lungs, and distant sites like the bone, brain, and peritoneum. Metastases are frequently present at diagnosis, indicating the systemic nature of the disease. Moreover, PDAC exhibits extensive molecular heterogeneity, with diverse genetic alterations driving tumor progression and treatment resistance. Mutations in genes such as KRAS, TP53, and SMAD4 contribute to PDAC’s aggressive phenotype, posing challenges for targeted therapy approaches. Last but not least, the PDAC tumor microenvironment is highly immunosuppressive and desmoplastic, facilitating tumor growth, invasion, and metastasis while impeding immune surveillance and therapeutic response. Stromal interactions and extracellular matrix remodeling further enhance tumor aggressiveness and therapeutic resistance (175–177).

Given PDAC’s aggressive nature and tendency for metastatic spread, comprehensive treatment strategies are essential. Although surgical resection holds promise for a treatment in cases of localized disease, the majority of patients are diagnosed with advanced-stage or metastatic PDAC, necessitating multidisciplinary approaches. These approaches should target not only the primary tumor but also metastases through systemic therapies, including chemotherapy, targeted agents, immunotherapy, and palliative interventions for symptom management. Moreover, recognizing PDAC’s systemic nature underscores the importance of early detection, accurate staging, and ongoing surveillance to tailor treatment appropriately. By addressing both the primary tumor and metastatic lesions, comprehensive treatment strategies aim to improve patient outcomes, prolong survival, and enhance quality of life despite the challenges posed by this aggressive malignancy (178–180).

## 5 Significance and management of bone metastases in advanced PDAC

Bone metastases pose a significant challenge in advanced PDAC, leading to a poorer prognosis and a diminished quality of life. They signify disease progression and are associated with adverse outcomes, causing skeletal complications such as pain, fractures, spinal cord compression, and hypercalcemia, severely impacting patient well-being. These events not only cause physical discomfort but also limit mobility and independence, compromising the overall quality of life. Furthermore, bone metastases indicate systemic disease spread, suggesting treatment resistance and limited therapeutic options. Managing bone metastases involves palliative measures to relieve pain and maintain skeletal integrity, including pain medications, bisphosphonates, denosumab, radiation therapy, and surgery. Despite these efforts, patients with bone metastases from PDAC often face a poor prognosis, with significantly lower survival rates than those without skeletal involvement. Hence, early detection and proactive management of bone metastases are crucial for improving patient outcomes and enhancing their quality of life in advanced PDAC. Addressing the challenges of treating bone metastases in PDAC requires innovative strategies. These challenges include late diagnosis, skeletal complications, treatment resistance, limited therapeutic options, and the heterogeneity of metastatic lesions. Emerging strategies involve targeted therapies, immunotherapy, radiopharmaceuticals, and combination treatments, offering hope for improving outcomes in this challenging scenario (181–183).

Zoledronic acid, a potent bisphosphonate medication, is widely used to manage bone metastases associated with various cancers. Administered intravenously, it targets the bone microenvironment, specifically inhibiting osteoclast-mediated bone resorption. Moreover, it binds to hydroxyapatite crystals in bone tissue, taken up by osteoclasts during bone resorption. It disrupts the mevalonate pathway within osteoclasts, inhibiting farnesyl pyrophosphate synthase, an important enzyme for the prenylation of small GTPase proteins. This impairs osteoclast function, halting bone resorption and the subsequent release of calcium ions and growth factors from the bone matrix. Zoledronic acid effectively reduces skeletal complications and improves clinical outcomes among patients afflicted with bone metastases arising from various cancers such as prostate cancer, breast cancer, and multiple myeloma. Clinical trials demonstrate its ability to decrease skeletal-related events (SREs) such as spinal cord compression, fractures, hypercalcemia, and the requirement for bone radiation or surgery. It also preserves bone mineral density and reduces bone pain, thereby enhancing patients’ quality of life. This compound has always been a valuable therapeutic option for managing bone metastases in PDAC, focusing on addressing skeletal-related events (SREs), relieving bone pain, and enhancing patients’ overall quality of life. Despite not directly targeting PDAC, integrating zoledronic acid into treatment plans for metastatic PDAC can significantly improve patient outcomes and quality of life by reducing symptoms and complications associated with bone metastases (184–188).

Emerging research and clinical trials are investigating the potential synergistic effects of combining zoledronic acid with other therapeutic modalities in managing advanced PDAC. While zoledronic acid primarily targets skeletal complications associated with bone metastases, its combination with other treatments aims to address the multifaceted nature of PDAC progression and enhance patient outcomes. In the first step, preclinical studies suggest potential synergistic effects between zoledronic acid and chemotherapy agents like gemcitabine and nab-paclitaxel. These combinations aim to target the primary tumor and bone metastases simultaneously, mitigating the risk of skeletal-related events. Ongoing clinical trials are evaluating the safety and effectiveness of integrating zoledronic acid with chemotherapy regimens in advanced PDAC cases. Moreover, combining zoledronic acid with immune checkpoint inhibitors or other immunotherapeutic agents aims to enhance antitumor immune responses and overcome immunosuppressive mechanisms in the tumor microenvironment. Preclinical studies investigating the immunomodulatory effects of zoledronic acid and its synergy with immunotherapy in PDAC are ongoing, with the goal of improving treatment responses and patient survival. Combining zoledronic acid with targeted therapies against molecular targets implicated in PDAC pathogenesis, on the other hand, may offer complementary mechanisms of action and enhance treatment efficacy. For example, combining zoledronic acid with inhibitors of the RAS-RAF-MEK-ERK pathway or the PI3K-AKT-mTOR pathway is being explored in clinical trials to overcome treatment resistance and improve outcomes in PDAC individuals suffering from bone metastases (185, 187–190).

Overall, while the combination of zoledronic acid with other therapeutic modalities shows promise in improving outcomes for advanced PDAC, addressing the challenges posed by bone metastases requires a comprehensive and multidisciplinary approach. Collaborative research efforts and innovative clinical trials are striving to optimize treatment strategies by exploring the synergistic effects of zoledronic acid combinations. However, it’s essential to recognize that a one-size-fits-all methodology is inadequate in the complex landscape of metastatic PDAC. Therefore, a multifaceted strategy encompassing targeted therapies, supportive care, and innovative treatment modalities is necessary to address the individual needs of patients and enhance their quality of life. By integrating zoledronic acid with tailored treatment regimens and considering patient-specific factors, healthcare professionals can strive towards more effective management of PDAC and its associated complications, ultimately leading to improved outcomes and better quality of life for patients (184, 191).

Comprehensive approaches to managing bone metastases in PDAC should encompass a range of strategies tailored to address the multifaceted nature of the disease. Firstly, targeted therapies directed against key signaling pathways implicated in PDAC progression and bone metastasis formation offer promise for improving treatment efficacy and overcoming resistance mechanisms. Combining targeted therapies with conventional treatments such as chemotherapy or immunotherapy may enhance therapeutic responses and prolong survival in PDAC patients with bone metastases. Secondly, symptom management and supportive care are essential components of comprehensive treatment approaches for PDAC patients with bone metastases. Palliative interventions such as pain management, physical therapy, and psychosocial support play a crucial role in alleviating symptoms, improving quality of life, and addressing the holistic needs of patients and their families. Lastly, ongoing research efforts are exploring novel treatment modalities and combination strategies to address the challenges posed by bone metastases in PDAC. This includes investigating the synergistic effects of zoledronic acid with chemotherapy, immunotherapy, or targeted agents, as well as exploring emerging therapies such as radiopharmaceuticals, tumor-targeted nanoparticles, and bone-targeted agents (185, 192–194).

By adopting a comprehensive approach that integrates targeted therapies, supportive care, and innovative treatment modalities, outcomes for PDAC patients with bone metastases can be optimized. Through collaborative research endeavors and patient-centered care, efforts are made to improve treatment responses, prolong survival, and enhance the overall quality of life for individuals affected by this devastating disease (195).

Moving forward, several key future directions in research and clinical practice for metastatic PDAC offer promise in advancing treatment options and enhancing patient outcomes. Continued exploration of novel therapeutic agents targeting molecular pathways implicated in PDAC progression and bone metastasis formation is essential. Additionally, personalized treatment strategies based on genomic profiling and molecular characterization hold potential for optimizing treatment selection. Integration of zoledronic acid into evolving therapeutic paradigms, along with the development of novel therapeutic approaches targeting bone metastases, presents exciting avenues for improving outcomes in PDAC patients. Embracing innovation and collaboration will be critical in advancing pancreatic cancer care and improving outcomes for individuals affected by this challenging disease (184, 190, 191).

## 6 Challenges in PDAC: the role of targeted therapies in overcoming treatment resistance

The clinical landscape of PDAC presents significant challenges, primarily stemming from late-stage diagnosis and limited treatment options, which contribute to its status as one of the most lethal malignancies. PDAC often progresses asymptomatically in its early stages, resulting in delayed diagnosis until the disease has advanced or metastasized. Consequently, only a minority of patients qualify for potentially curative surgical resection, while the majority are diagnosed with locally advanced or metastatic disease (196, 197).

In the clinical landscape of PDAC, several challenges persist. Firstly, PDAC is frequently diagnosed at advanced stages, marked by extensive local invasion or distant metastasis, greatly limiting treatment options and reducing the likelihood of long-term survival. Furthermore, the therapeutic arsenal available for PDAC is relatively constrained compared to other cancer types. While surgery, chemotherapy, and radiation therapy remain the mainstays of treatment, their efficacy in advanced-stage disease is modest, with low response rates and limited survival benefits. Additionally, PDAC displays a notorious resistance to conventional therapies, including chemotherapy and radiation. Tumor heterogeneity, the desmoplastic tumor microenvironment, and molecular alterations contribute to this resistance, posing significant challenges in achieving durable responses and disease control. Lastly, despite advancements in cancer treatment, PDAC continues to have a dismal prognosis, with five-year survival rates remaining below 10%. The aggressive nature of the disease, combined with its late-stage diagnosis and restricted treatment options, emphasizes the urgent need for innovative therapeutic approaches to enhance patient outcomes (196–198).

In this challenging clinical scenario, targeted therapies offer a promising avenue for addressing the molecular drivers of PDAC and overcoming treatment resistance. Targeted agents directed against specific molecular pathways implicated in PDAC pathogenesis, such as the RAS-RAF-MEK-ERK and PI3K-AKT-mTOR pathways, hold potential for improving treatment efficacy and patient outcomes. By targeting key signaling pathways aberrantly activated in PDAC, these therapies aim to disrupt tumor growth, induce apoptosis, and enhance sensitivity to conventional treatments. However, the development of effective targeted therapies for PDAC has been hindered by the complex molecular landscape of the disease and the lack of easily targetable molecules. Challenges such as tumor heterogeneity, acquired resistance, and off-target effects necessitate a comprehensive understanding of PDAC’s underlying biology and the identification of predictive biomarkers to guide treatment selection. Despite these challenges, ongoing research efforts in targeted therapy development offer hope for improving outcomes in PDAC. By elucidating the molecular mechanisms driving tumor growth and metastasis, targeted therapies have the potential to revolutionize the treatment landscape of PDAC and provide new avenues for personalized and precision medicine approaches (199–201).

## 7 Precision oncology approaches in PDAC: current landscape and future perspectives

Despite decades of research and therapeutic advancements in other cancer types, the management of PDAC remains a formidable challenge. Precision oncology has emerged as a promising paradigm shift in cancer treatment, offering tailored therapeutic strategies based on the molecular characteristics of individual tumors. In this comprehensive review, we delve into the current state of treatment and therapies for PDAC from a precision oncology perspective, exploring genomic profiling, targeted therapies, immunotherapy, personalized treatment strategies, challenges, and future directions (6).

Genomic profiling serves as the cornerstone of precision oncology in PDAC. Through the advent of high-throughput sequencing technologies, comprehensive characterization of the genomic landscape of pancreatic tumors has become feasible. Notably, mutations in key driver genes such as KRAS, TP53, CDKN2A, and SMAD4 are commonly observed, offering potential therapeutic targets. Additionally, molecular subtyping based on gene expression profiles has unveiled distinct subtypes of PDAC with unique biological behaviors and therapeutic susceptibilities. Integration of genomic data into clinical decision-making holds promise for optimizing treatment selection and improving patient outcomes. Moreover, genomic profiling in PDAC not only encompasses the analysis of protein-coding genes but also involves the exploration of non-coding regions of the genome, including ncRNAs. Through high-throughput sequencing technologies, researchers can identify alterations in ncRNA expression profiles, such as dysregulated miRNAs or lncRNAs, which may serve as diagnostic or prognostic biomarkers. Furthermore, molecular subtyping based on ncRNA expression patterns can contribute to the classification of distinct PDAC subtypes with unique therapeutic susceptibilities (202–204).

Targeted therapies aim to exploit specific molecular vulnerabilities within cancer cells while minimizing systemic toxicity. In PDAC, aberrant activation of signaling pathways such as the MAPK and phosphatidylinositol 3-kinase (PI3K)/AKT/mTOR pathways offers avenues for targeted intervention. Clinical trials investigating targeted agents, including MEK inhibitors and PI3K pathway inhibitors, have demonstrated promising results in preclinical models and early-phase trials. However, challenges such as tumor heterogeneity and acquired resistance underscore the need for further research to optimize targeted therapy approaches in PDAC. In the search for targeted therapies for PDAC, researchers are investigating the molecular vulnerabilities conferred by dysregulated ncRNAs. For instance, aberrant expression of specific miRNAs or lncRNAs may activate oncogenic signaling pathways, such as the MAPK or PI3K/AKT/mTOR pathways, driving tumor growth and progression. Targeting these dysregulated ncRNAs with therapeutic agents, such as miRNA mimics or inhibitors, holds promise for disrupting oncogenic signaling and inhibiting tumor growth (199–201).

There is little room for doubt that immunotherapy has revolutionized cancer treatment across various malignancies but has shown limited success in PDAC. The immunosuppressive tumor microenvironment, characterized by dense stromal desmoplasia and immune evasion mechanisms, poses significant challenges to effective immunotherapy. Despite this, ongoing efforts aim to enhance the efficacy of immunotherapy in PDAC through combination strategies, including adoptive cell therapies, cancer vaccines, and immune checkpoint inhibitors. Biomarker discovery and patient stratification are critical for identifying individuals likely to benefit from immunotherapy and overcoming resistance mechanisms. Newly emerging findings suggest that ncRNAs may influence the immune response within the tumor microenvironment. For example, certain miRNAs may regulate the expression of immune checkpoint molecules, influencing the efficacy of immunotherapy. Understanding the interplay between ncRNAs and the immune response in PDAC is critical for optimizing immunotherapeutic strategies (205–208).

Personalized treatment strategies in PDAC encompass the integration of genomic profiling, biomarker assessment, and clinical data to tailor therapy to individual patients. Liquid biopsy techniques, such as circulating tumor DNA analysis, offer non-invasive means for monitoring disease progression and detecting treatment-resistant mutations. Furthermore, the identification of predictive biomarkers, such as homologous recombination deficiency (HRD) status, guides the selection of patients who may benefit from specific therapies, including platinum-based chemotherapy and PARP inhibitors. The evolution towards personalized medicine holds promise for optimizing treatment efficacy while minimizing treatment-related toxicity in PDAC patients. It is to be noted that personalized treatment strategies in PDAC extend beyond genomic profiling to include the assessment of ncRNA expression profiles. Liquid biopsy techniques, such as circulating miRNA or lncRNA analysis, offer non-invasive methods for monitoring disease progression and predicting treatment response. Moreover, the identification of predictive biomarkers among ncRNAs, such as those associated with chemoresistance or tumor aggressiveness, enables clinicians to tailor therapy for each patient, optimizing treatment effectiveness while curtailing potential side effects (204, 209–211).

Despite significant advancements, several challenges impede the widespread implementation of precision oncology in PDAC. These include the need for comprehensive genomic profiling infrastructure, the limited availability of targeted agents, and the development of resistance mechanisms to therapy. Future research directions may involve the exploration of novel therapeutic targets, the development of rational combination treatment approaches, and the integration of multi-omics data to enhance treatment response prediction. Collaborative efforts between clinicians, researchers, and industry stakeholders are essential to address these challenges and translate scientific discoveries into tangible clinical benefits for PDAC patients. Precision oncology represents a paradigm shift in the management of PDAC, offering personalized therapeutic approaches tailored to the molecular characteristics of individual tumors. Genomic profiling, targeted therapies, immunotherapy, and personalized treatment strategies stand as promising avenues for improving patient outcomes in this devastating disease. Despite existing challenges, continued research efforts hold the potential to overcome barriers and usher in a new era of precision medicine for PDAC (199, 212–216).

## 8 Conclusion and perspective

**Figure 5:**
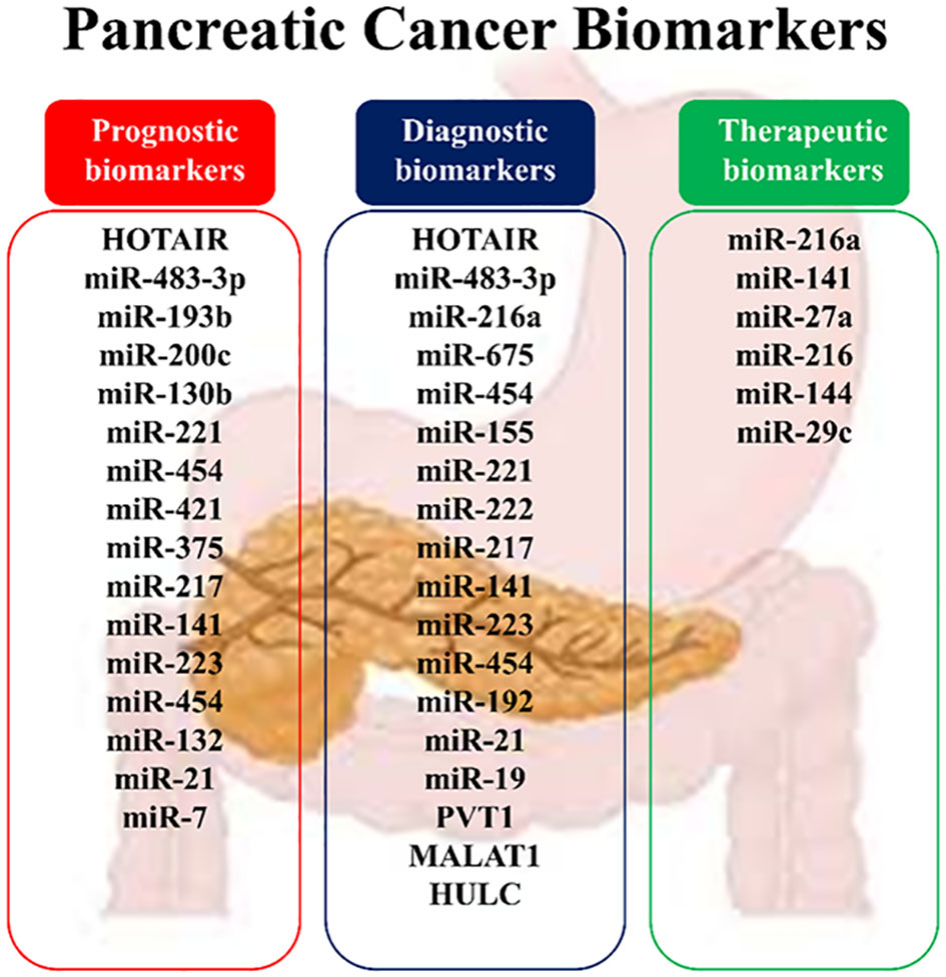
Noncoding RNAs as biomarkers in pancreatic cancer.

PDAC poses a paramount global health challenge given its aggressive nature, dismal prognosis, and limited treatment options. With a median life expectancy of less than six months, advancements in diagnostic and therapeutic strategies are urgently needed. ncRNAs, a substantial portion of the human genome, are implicated in cancer development and progression, including pancreatic carcinoma. This study explores the roles of specific ncRNAs, particularly lncRNAs and miRNAs, in PDAC, focusing on their involvement in tumor growth, chemoresistance, and prognosis. Biomarker development is crucial for managing PDAC, aiding in early diagnosis, treatment selection, and prognosis assessment (**Figure 5**). Changes in ncRNA expression throughout PDAC development signify alterations in cellular signaling pathways, highlighting their potential as therapeutic targets. While research on lncRNAs and miRNAs presents challenges due to their size and complexity, their functions in PDAC pathogenesis have been recognized, with miRNAs showing particular promise due to their ease of sequencing and functional analysis. However, understanding the precise biological functions of ncRNAs remains incomplete, hindering an accurate interpretation of their roles. Deciphering the regulatory mechanisms of ncRNAs, particularly the stimuli driving their organization in specific cellular compartments, presents challenges (31, 68, 93).

To address these limitations, future studies could employ advanced techniques such as CRISPR/Cas9-based gene editing for in-depth functional characterization of lncRNAs. Investigating the stimuli influencing the organization of lncRNAs and integrating multi-omics approaches may provide a more comprehensive understanding of ncRNA networks in PDAC. Large-scale validation studies are essential to assess the clinical utility of ncRNAs as biomarkers and translate these findings into practical applications, such as targeted therapies and diagnostic tools. Overcoming these challenges and implementing suggested improvements will enhance our understanding of ncRNA involvement in PDAC, ultimately leading to improved patient outcomes (157–159).

## Author contributions

SJ: Supervision, Writing – original draft. HM: Writing – original draft. PJ: Supervision, Writing – original draft. KJ: Writing – original draft. AM-A: Writing – review & editing. AA: Supervision, Writing – review & editing. GA: Writing – review & editing.
